# Alexithymic Traits and Hair Cortisol Concentrations in Pregnant Women

**DOI:** 10.3389/fpsyt.2020.00421

**Published:** 2020-05-13

**Authors:** Jani Kajanoja, Max Karukivi, Paula Mustonen, Noora M. Scheinin, Susanna Kortesluoma, Ana João Rodrigues, Hasse Karlsson, Linnea Karlsson

**Affiliations:** ^1^ Department of Psychiatry, University of Turku and Turku University Hospital, Turku, Finland; ^2^ Department of Psychiatry, Satakunta Hospital District, Pori, Finland; ^3^ FinnBrain Birth Cohort Study, Population Health Research Centre, University of Turku, Turku, Finland; ^4^ Department of Adolescent Psychiatry, Satakunta Hospital District, Pori, Finland; ^5^ Department of Child Psychiatry, University of Turku and Turku University Hospital, Turku, Finland; ^6^ School of Medicine, Life and Health Sciences Research Institute (ICVS), University of Minho, Braga, Portugal

**Keywords:** alexithymia, stress, cortisol, prenatal, emotional regulation, psychosomatic medicine

## Abstract

**Introduction:**

Alexithymia, a personality construct characterized by difficulties in identifying and expressing emotions, and an externally oriented thinking style, has been associated with a number of stress-related disorders, and physiological markers of stress. We examined the relationships of alexithymia and hair cortisol concentrations (HCC), a measure of long-term cortisol levels, in pregnant women.

**Methods:**

Participants were 130 women from the FinnBrain Birth Cohort study. Alexithymia was measured with the Toronto Alexithymia Scale (TAS-20). Analysis of covariance and regression analyses were used to assess the associations between alexithymia and HCC. Educational level, current depressive symptoms, and body mass index (BMI) were applied as covariates.

**Results:**

In the adjusted analyses, individuals with moderate to high alexithymic traits had significantly higher HCC (*F* = 5.11, partial *η*² = 0.040 , *p* = 0.026) compared to non-alexithymics. Regression analyses in the whole sample revealed that, of the individual dimensions of alexithymia, Difficulty Identifying Feelings (DIF) was associated with HCC (β = 0.187, *t* = 2.064, *p* = 0.041).

**Conclusions:**

Alexithymia, and especially its dimension DIF, were associated with higher HCC and, therefore, may be linked to increased chronic physiological stress. Implications for pregnancy outcomes and infant development are discussed.

## Introduction

Alexithymia is a personality construct characterized by difficulties in identifying and expressing emotions, and an externally oriented thinking style, lacking tendencies for fantasy and imagination (1). Although it is not considered a psychiatric disorder in itself, alexithymia has been linked with a number of stress-related disorders, such as depression, anxiety, pain syndromes, and an increased risk of cardiovascular disease and mortality (2–6). According to the stress-alexithymia hypothesis, alexithymic individuals, due to a lack of emotional awareness, may fail to identify and respond to stressful events adequately, thus leading to increased levels of chronic stress (7). This chronic stress in turn could explain the higher psychiatric and somatic morbidity associated with alexithymia.

Previous studies have provided support for the stress-alexithymia hypothesis, showing increased cortisol responses in alexithymic individuals following a social stress test (8, 9). As for chronic stress measures, alexithymia has been linked to increased cortisol secretion in the dexamethasone suppression test, as well as an aberrant cortisol awakening response (10, 11). Furthermore, several studies have associated alexithymia to altered immune responses and tissue inflammation, possibly reflecting physiological effects of prolonged stress (12–14). Interestingly, alexithymia seems also to be more prevalent in patients with immune-related disorders, such as rheumatoid arthritis, multiple sclerosis, and systemic lupus erythematosus (15–17). Existing studies on alexithymia and stress have largely focused on acute stress markers in experimental settings and have rarely controlled for the effects of depressive symptoms, which are highly prevalent in alexithymic individuals (3).

To our knowledge, only two studies have investigated alexithymia during pregnancy (18, 19). In both studies alexithymia was related to psychiatric symptoms during pregnancy. Le et al. (18) additionally concluded that alexithymia was a stable phenomenon during pregnancy and the postnatal period. No studies have investigated the association of alexithymia and objective stress measures during pregnancy. Elucidating psychological factors associated with physiological stress is especially relevant in the prenatal context, as psychosocial stress during the gestational period can have long-term consequences for offspring development and future health outcomes (20). Glucocorticoids are able to partially pass the placental barrier and play an important role in fetal development. However, excessive exposure to glucocorticoids may have adverse consequences for the developing brain. Animal models have indicated that prenatal stress or glucocorticoid treatment induces permanent changes in offspring physiology that increase the risk for later somatic and neuropsychiatric disease (21–23). Similarly, in humans, elevated maternal glucocorticoid levels may disrupt fetal brain development and harmfully affect child socioemotional functioning (20, 24).

The aim of this study was to test the stress-alexithymia hypothesis by analyzing the association of alexithymia, depressive symptoms, and physiological stress in the prenatal period. We examined alexithymia levels and hair cortisol concentrations (HCC) in a birth cohort sample of pregnant women. Hair cortisol is an emerging potential biomarker for chronic stress (25, 26) and is thought to reflect cumulative cortisol concentrations over the previous months (27). According to the stress-alexithymia hypothesis, we expected higher alexithymia levels to be associated with higher HCC.

## Methods

### Study Details and Participants

This study is based on the FinnBrain Birth Cohort study (www.finnbrain.fi), a prospective cohort established to study the effects of prenatal and early life stress exposure on child brain development and health (28). Participants were recruited between December 2011 and April 2015 from maternal welfare clinics in the South-Western Hospital District and the Åland Islands in Finland. After recruitment, the participants filled in a set of self-report questionnaires three times during pregnancy, at gestational weeks (gwk) 14, 24, and 34. After birth, the families are followed up at 3- to 6-month intervals (the first 30 months) or 12-to 36-month intervals (from 36 months onward), and the study is planned to continue for decades. The parents gave written informed consent on their own and on their child’s behalf. The children will be asked for personal consent at an appropriate age. The ethics committee of the Hospital District of Southwest Finland has approved the study protocol (number of ethical approval ETMK-57/180/2011). The participants for this study consist of those mothers who provided hair samples (collection began at the end of the cohort recruitment) and filled in all relevant questionnaires concerning alexithymia levels and depressive symptoms (N = 130). Depressive symptoms were assessed in the 3rd trimester of pregnancy (at gwk 34), and hair samples were collected at the maternity ward 1 to 3 days after childbirth.

Educational level and BMI were considered as potential confounders as they have previously been associated with both alexithymia (29, 30), as well as HCC levels (31, 32). Depressive symptoms were also initially controlled for because the measurement of alexithymia may show some overlap with negative affect (33). However, consistently with previous research (31), depressive symptoms were not associated with HCC levels, and were therefore removed from the final analyses. Regarding possible substance abuse problems, our protocol was to refer participants with substance abuse to treatment. However, as discussed below, no participants reported problematic substance use during pregnancy.

## Questionnaire Data

Questionnaire data included a variety of background information on the participants. For this study, we included age, body mass index (BMI), previous, and/or current substance use (including alcohol, tobacco, and illicit drugs), and the level of education divided into five classes: 1) high school or lower; 2) vocational degree; 3) upper secondary school; 4) applied sciences or bachelor’s degree; 5) graduate school or PhD degree. Current medication use during pregnancy was inquired including antidepressant (SSRI or SNRI), non-steroidal anti-inflammatory (NSAID), and glucocorticoid and thyroid medication use. Background information was collected in the 1st trimester of pregnancy, substance use was additionally assessed in the 3rd trimester. Participants were also asked to report any diagnosed medical or psychiatric conditions.

Toronto Alexithymia Scale (TAS-20) (34, 35) is one of the most commonly used self-report scales used to measure alexithymic features. It consists of 20 items divided into three subscales: difficulty identifying feelings (DIF), difficulty describing feelings (DDF), and externally oriented thinking style (EOT). Items are rated with a five-point Likert scale (1, strongly disagree; 5, strongly agree). Thus, the total score ranges from 20 to 100. An individual is considered highly alexithymic if the TAS-20 total score exceeds 60 points, and moderately alexithymic if the total score is between 52 and 60 points (36). The TAS-20 was administered 6 months after the baby was born. Alexithymia levels were assessed both continuously as well as categorically. As the prevalence of high alexithymia was very low (3.1%, N = 4), the cutoff score of moderate to high alexithymia (TAS-20 overall score > 52) was used. Individuals scoring under 52 points were classified as non-alexithymic.

The Edinburgh Postnatal Depression Scale (EPDS) (37) is a widely used questionnaire for screening prenatal and postnatal depression. It is a 10-item self-report scale that asks respondents to rate their mood and other depressive symptoms in the previous 7 days. Questions are scored from 0 to 3, and thus, the total score ranges from 0 to 30 points. Depressive symptoms were measured at gwk 34 and used as a continuous variable.

### Hair Cortisol Assessment

Maternal hair samples were collected from a random population of the FinnBrain Cohort participant mothers at the maternity ward 1 to 3 days after delivery between December 2014 and November 2015. A strand of hair was cut from a standardized area of the posterior vertex region of the head as close to the scalp as possible. Hair samples were stored in foil in a dry place protected from light according to good research practice, Finnish legislation, and data protection until the analyses. The analyses were performed at Life and Health Sciences Research Institute (ICVS), University of Minho, Portugal. For the analysis, a 5-cm segment was used. As hair grows approximately 1 cm per month (38), a 5-cm segment was estimated to reflect the cortisol concentrations of the previous 5 months.

The hair segments were washed in isopropanol for 3 min three times and finely minced using surgical scissors. For extraction of cortisol, 1.5 ml of methanol was added to each sample, and the samples were incubated at 55°C for 24 h. After centrifuging at 10,000 rpm for 2 min, the supernatant was transferred to a new vial. Methanol was evaporated at 60°C under a constant stream of nitrogen until the samples were dried completely. Finally, 0.15 ml of phosphate buffer was added, and 50 μl of each sample was analyzed with ELISA (IBL International Cortisol Saliva ELISA) following the manufacturer’s procedure. All samples were analyzed in duplicates with coefficients of variation below 15%.

The HCC data were examined for outliers, and values >3 standard deviations (SD) above the mean were excluded from the final analyses (HCC > 190.5 pg/mg, N = 5) (31). Hair samples weighing < 5 mg (N = 0) or > 15 mg (N = 7) were excluded.

### Statistical Methods

All statistical analyses were conducted using the IBM SPSS (version 24.0). Normality of distribution within variables was assessed with the Shapiro-Wilk test. Associations between categorical variables were analyzed with Chi Square test. Student’s t-test was used to examine differences in HCC between groups. As BMI was non-normally distributed, Mann Whitney U-test was used. An analysis of covariance (ANCOVA) was conducted to examine group differences in HCC between alexithymic individuals and non-alexithymics while controlling for the selected covariates. Multiple regression analyses were conducted to examine the associations between alexithymia dimensions, overall alexithymia scores, and HCC, controlling for the effects of educational level, BMI, and current depressive symptoms (EPDS) at gwk 34. As substance use levels were negligible, they were not used as covariates. Maternal age was unrelated to both alexithymia levels and HCC, and was thus also left out of the final analyses. Natural logarithmic transformations were performed on the HCC data to reduce skewness according to common practice (26, 39). After transformation, HCC was normally distributed (Shapiro-Wilk test, *p* > 0.4 for both groups).

## Results

Basic information on the study sample is provided in **Table 1**. Only one participant in each group reported tobacco use after the 1st trimester of pregnancy. Nine individuals (7.7%) in the non-alexithymia group, and one (8.3%) in the moderate to high alexithymia group reported alcohol use after the 1st trimester. Frequency of use in all cases was less than once a month, and ≤ 1 standard dose of alcohol per occasion. No participants reported current illicit drug use. Three participants reported SSRI/SNRI use during pregnancy, eight participants reported thyroid medication (levothyroxine) use during pregnancy, and five participants reported glucocorticoid use during pregnancy. None of the participants reported NSAID use. Regarding somatic diseases and disorders potentially affecting the immune system or causing inflammation, one participant reported a diagnosed cancer and two reported a diagnosis of an autoimmune disorder (not further specified). No one reported current bacterial or viral infections. Mean duration of pregnancy was 280 days (min, 254 days; max, 296 days). Duration of pregnancy had no correlation with HCC (Spearman’s rho = 0.032, *p* = 0.714), TAS-20 scores (rho = 0.117, *p* = 0.185), or DIF (rho = 0.116, *p* = 0.189).

**Table 1:** Basic information and comparison of hair cortisol concentrations between alexithymia groups.

	No alexithymia (N = 118)	Moderate to high alexithymia (N = 12)	Test statistic	*p*
Level of education* 1. 2. 3. 4. 5.	0% 9.6% 9.6% 37.4% 43.5%	16.7% 25.0% 8.3% 25.0% 25.0%	−2.257^b^	**0.024**
Age	Mean (SD) 31.6 (3.7)	Mean (SD) 30.2 (4.6)	1.24^a^	0.217
Depressive symptoms	4.3 (3.9)	7.3 (3.9)	−2.55^b^	**0.011**
BMI	24.2 (27.6)	27.6 (7.2)	−1.68^b^	0.094
HCC (pg/ml)	17.8 (19.3)	35.1 (31.5)	−2.73^a^	**0.007**
DIF	11.2 (3.6)	20.8 (4.3)	−5.22^b^	**<0.001**
DDF	9.2 (3.0)	16.4 (3.4)	−5.12^b^	**<0.001**
EOT	17.7 (4.3)	22.0 (3.4)	−3.21^b^	**<0.001**
TAS-20 total	38.0 (7.2)	59.2 (5.8)	−5.67^b^	**<0.001**

a Student’s t-test.

b Mann Whitney U test.

*Level of education: 1, high school or lower; 2, vocational degree; 3, upper secondary school; 4, applied sciences or bachelor’s degree; 5, graduate school or PhD degree.

HCC, hair cortisol concentrations; BMI, body mass index; DIF, difficulty identifying feelings; DDF, difficulty describing feelings; EOT, externally oriented thinking.

No alexithymia = TAS-20 score ≤ 52.

Moderate to high alexithymia = TAS-20 score > 52.

**Figure 1:**
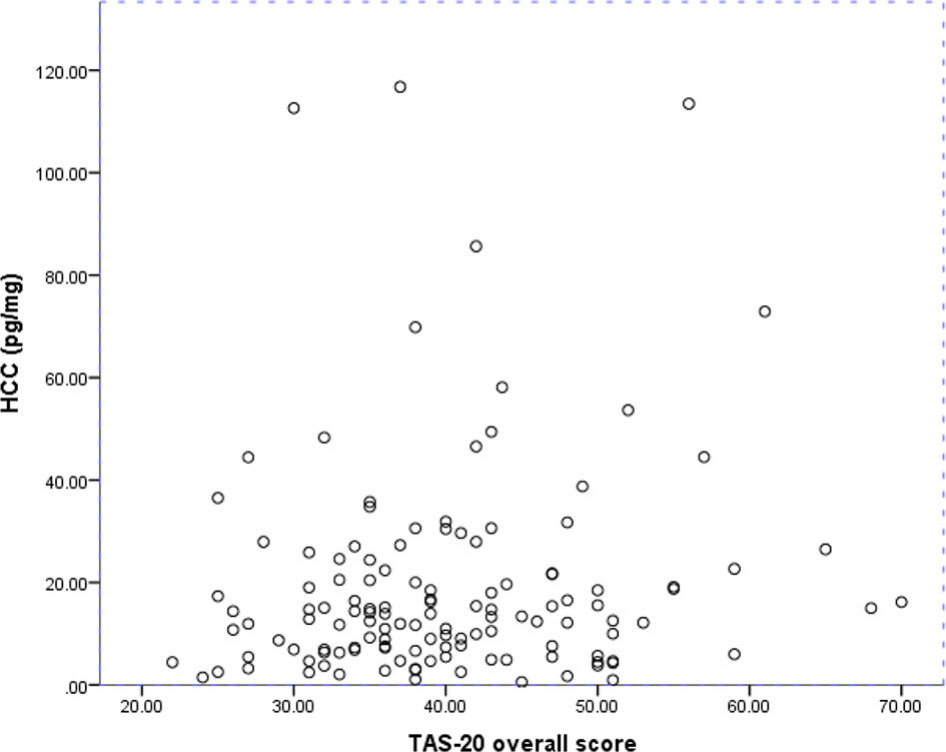
Hair cortisol concentrations and overall alexithymia levels in the whole sample. HCC, Hair cortisol concentrations. TAS-20, Toronto Alexithymia Scale.

**Figure 1** shows the distributions of HCC and alexithymia levels. The moderate/high alexithymia group showed higher HCC levels compared to those with low alexithymia levels (*t* = 2.75, *p* = 0.007). Results of the ANCOVA showed that the difference in HCC between alexithymia groups stayed significant after controlling for the selected covariates (*F* = 5.11, partial *η*² = 0.040, *p* = 0.026). One participant in each group reported SSRI/SNRI use during late pregnancy. Removing these participants from the analyses slightly strengthened the group difference (*F* = 6.12, partial η² = 0.049, *p* = 0.015). Additionally, one participant in the moderate/high alexithymia group reported SSRI/SNRI use only in the first trimester of pregnancy. Removing all three SSRI/SNRI users did not affect the results. Five participants in the low alexithymia group reported current glucocorticoid use, and removing these did not affect the results. Removing those with thyroid conditions/medications, cancer, and autoimmune disorders did not affect the results either.

Separate regression analyses were conducted for TAS-20 and its individual dimensions in the whole sample. As the EPDS score was not associated with the outcome variable HCC (Spearman’s rho = 0.092, *p* = 0.298), it was excluded from the covariates in the regression analyses.

After controlling for confounders, DIF remained a significant predictor of HCC (**Table 2**). The associations between HCC and TAS-20 total score, as well as the two other (DDF and EOT) alexithymia dimensions were non-significant (*p* > 0.1 for all comparisons). Of the covariates, educational level and BMI were negatively associated with HCC (**Table 2**).

**Table 2:** Summary of multiple regression analysis of variables predicting hair cortisol concentration.

	B	Standard error	β	t	p
Body mass index	−0.045	0.017	−0.243	−2.668	0.009
Level of education	−0.213	0.080	−0.234	−2.667	0.009
DIF	0.039	0.019	0.187	2.064	0.041

DIF, difficulty identifying feelings.

## Discussion

Our results suggest that, among pregnant women, moderate to high alexithymia is associated with elevated HCC levels, compared to the non-alexithymic group. This association was driven by subjective difficulty in identifying feelings (DIF), as it was the only dimension of alexithymia that was associated with HCC in the whole sample, after controlling for potential confounders. While educational level and BMI were both negatively associated with HCC, depressive symptom scores in late pregnancy (gwk 34) were unrelated to HCC.

Our results are in line with previous research that has associated alexithymia with altered HPA axis functioning, inflammatory markers, and immune responses (8–14, 39). A review article by De Berardis et al. (40) summarized findings showing that alexithymia has been consistently linked to higher circulating levels of acute phase proteins, as well as proinflammatory cytokines. They argued that alexithymia may involve a chronic hyperreactivity of the HPA axis to stressful situations, increasing cortisol levels, which in turn affect immune responses and cytokine production. As hair cortisol is considered a marker of long-term HPA axis activity, our findings lend support to these ideas. Chronically heightened cortisol concentrations as a result of prolonged psychosocial stress may at least partially explain the altered immune system functioning, as well as the higher prevalence of alexithymia in individuals suffering from stress- and immune-related disorders.

Several psychological mechanisms could explain the apparent link between alexithymia and physiological stress. Martin & Pihl (7), in their stress-alexithymia hypothesis, proposed that alexithymia could directly increase long-term stress levels due to an impaired ability to identify and downregulate stress responses. The fact that only DIF was related to increased hair cortisol levels is partially in line with previous research: In a study by Hua et al. (9), only the DIF dimension of alexithymia was associated with increased salivary cortisol secretion before, during, and after a social stress test. They suggested that DIF may impede emotional appraisal and may, therefore, increase anticipatory reactions to stressful situations and hinder recovery from them. A recent study by De Berardis et al. (41) found that DIF together with low resilience was associated with suicide ideation. They hypothesized that alexithymia, and particularly the DIF dimension may contribute to low resilience *via* maladaptive coping strategies. In sum, from the different facets of alexithymia, DIF may be particularly relevant for stress vulnerability and resilience.

However, other mediating factors for the association of alexithymia and stress are also possible. Alexithymic individuals tend to suffer from social anhedonia and interpersonal difficulties, possibly making everyday social situations more stressful. Alexithymic individuals are also more prone to substance use, and more often engage in a sedentary lifestyle and unhealthier diets (42–44), all of which may increase physiological stress levels. As our study was cross-sectional and did not assess potential mediating factors for chronic stress, future studies will need to address specific causal pathways between alexithymia and stress. Furthermore, as prenatal stress in mothers may have programming effects for fetal development, increasing the likelihood of future somatic and psychiatric morbidity in the offspring, future studies should examine the effects of maternal alexithymia on child development.

Some limitations of this study should be considered. The prevalence of alexithymia in this sample, as well as in the whole birth cohort, was low compared to the prevalence of alexithymia in the general population (45, 46), and the distribution of HCC was substantially wide. The low prevalence of alexithymia may be explained by the fact that the FinnBrain study sample is relatively highly educated and consists of young adults, thus the results may not be generalizable to all populations and other countries. Therefore, the results should be replicated in a larger and more representative sample of alexithymic individuals, and with more diverse measures of stress. Furthermore, HPA axis metabolism as measured by HCC in the context of alexithymia should be investigated among non-pregnant females and males, to gain more generalizable knowledge on the associations between long-term HPA axis homeostasis and alexithymia. Another limitation was that alexithymia was measured postnatally, whereas HCC was measured during late pregnancy. Cortisol levels are generally known to rise toward the end of pregnancy, and little longitudinal data exists on postnatal alterations off HCC (47). However, this is a minor limitation as several studies have concluded that alexithymia is a relatively stable trait (48), including in the course of pregnancy and the postnatal period (18). Finally, we did not assess traumatic experiences in adult life, adjustment disorders or post-traumatic stress disorder, and therefore we cannot speculate whether the alexithymic features in our study sample were attributable to developmental factors, traumatic experiences in adulthood or some other predisposing factor.

## Conclusions

Alexithymia in pregnant women was associated with higher hair cortisol concentrations during the third trimester, possibly indicating chronic prenatal stress. The association was driven by subjective difficulty in identifying feelings, and was independent of current depressive symptoms. Further research is warranted to examine if alexithymia plays a causal role in prenatal physiological stress, and whether maternal alexithymia may affect offspring development.

## Data Availability Statement

The datasets generated for this study are available on request to the corresponding author.

## Ethics Statement

The studies involving human participants were reviewed and approved by Ethics Committee, Hospital District of Southwest Finland. Written informed consent to participate in this study was provided by the participants’ legal guardian/next of kin.

## Author Contributions

All authors have been involved in the writing process of the article. JK, MK, NS, LK, and HK were involved in planning the research. JK has conducted the statistical analyses and most of the planning and writing.

## Funding

We wish to thank the Finnish Cultural Foundation Satakunta Regional Fund, Signe, and Ane Gyllenberg Foundation, Turku University Foundation, Jane and Aatos Erkko Foundation, and Academy of Finland (Grant number 287908) for funding this study. In addition, the FinnBrain Birth Cohort Study has received funding for this part of the project from the Academy of Finland (grants 134950 and 253270) and Finnish State Grants for Clinical Research (ERVA projects P3003, P3498, and P3654).

## Conflict of Interest

The authors declare that the research was conducted in the absence of any commercial or financial relationships that could be construed as a potential conflict of interest.
